# Prevalence and Predictors of Irritable Bowel Syndrome (IBS) Among Medical Students at the University of Sharjah, UAE

**DOI:** 10.7759/cureus.71758

**Published:** 2024-10-18

**Authors:** Amin Fraij, Anood Shukry, Abdullah Omira, Joudi Habbal, Mariam Al Ali, Noor Jamal, Sura Alawsi, Iman Talaat

**Affiliations:** 1 College of Medicine, Emirates Health Services, Sharjah, ARE; 2 College of Medicine, Dubai Health, Dubai, ARE; 3 College of Medicine, Department of Health, Al Ain, ARE; 4 College of Medicine, Department of Health, Abu Dhabi, ARE; 5 Clinical Sciences, University of Sharjah, Sharjah, ARE

**Keywords:** ibs (irritable bowel syndrome), medical students, predictors, prevalence, united arab emirates (uae)

## Abstract

Background

Irritable bowel syndrome (IBS) is the most common functional gastrointestinal disorder (FGID), with different subtypes based on symptoms. Psychological stress has been found to have a significant impact on intestinal function and is associated with self-reported IBS and gastrointestinal symptoms.

Aims

This study aimed to determine the prevalence of irritable bowel syndrome (IBS) among medical students at the University of Sharjah in the United Arab Emirates and to identify potential predictors of IBS.

Methods

A cross-sectional study was conducted in 2019 at the University of Sharjah. A multistage stratified random sampling technique was employed to recruit undergraduate students from medicine, dentistry, pharmacy, and health sciences colleges. A questionnaire consisting of 27 questions was administered to 471 medical students, which incorporated both standardized tools (Perceived Stress Scale (PSS-4), Hospital Anxiety and Depression Scale (HADS), and Rome IV criteria) and custom-developed sections (demographics and food frequency questionnaire). The data collected was analyzed using descriptive and bivariate statistics. The level of significance was set at 5% (p < 0.05).

Results

The research found that a moderate prevalence (17.3%) of IBS was present among medical students at UOS, with stress, anxiety, and smoking being the most significant predictors. No significant correlation was found between the occurrence of IBS and both gender and dietary factors.

Conclusion

The study identified a moderate prevalence (17.3%) of IBS among medical students, in which stress, anxiety, and smoking were the most significant predictors. Screening programs for IBS and psychological problems are recommended, along with stress coping strategies courses and raising awareness among the general population about IBS. The study also suggests conducting similar studies among the general population in the UAE to determine the prevalence of IBS and identify potential risk factors.

## Introduction

Functional gastrointestinal disorder (FGID) is a term used to describe a variety of chronic or recurring gastrointestinal symptoms without an identifiable underlying cause [1]. Research from 2014 found that more than a third of new patient referrals to a gastroenterology clinic were diagnosed with FGID, with inflammatory bowel syndrome (IBS) being the most common [2]. IBS is associated with significant impairments in daily functioning and reduced health-related quality of life [3]. It is characterized by abdominal pain, diarrhea and/or constipation, mucus passing along with stools, and changes in stool form [4]. Since IBS falls under the FGID umbrella, its etiology is not well established yet [1]. Sufferers of IBS do not exhibit any observable structural irregularities to account for their symptoms [5,6]. The absence of definitive biomarkers or indicators presents a challenge in establishing precise diagnostic criteria, and diagnosis is primarily reliant on clinical assessment [7].

In different countries and studies, the prevalence of irritable bowel syndrome (IBS) varies considerably. A 2012 meta-analysis found a worldwide pooled prevalence of 11.2%, ranging from 1.1% to 45.0% depending on the country [8]. There is limited data on the prevalence of IBS in the Middle East, with studies conducted mainly in Saudi Arabia and Lebanon [9,10]. However, there is a lack of information regarding the prevalence of IBS in the United Arab Emirates (UAE). Numerous factors have been associated with IBS, including gender, age, family history, and psychological issues [11]. Clinical and experimental evidence suggests that psychological stress significantly affects intestinal function [12]. Medical school is commonly perceived as highly stressful and demanding, and this perception is reflected in higher stress levels among medical students compared to the general population [13,14]. A study conducted among medical students at King Abdulaziz University in Jeddah, Saudi Arabia, reported a prevalence of IBS at 31.8% [15].

To address the significant lack of available data in the UAE regarding irritable bowel syndrome (IBS), this study was conducted specifically among medical students at the University of Sharjah. This demographic is particularly relevant due to the high levels of stress and anxiety they experience during their training, which may influence the prevalence of IBS. Previous research has primarily focused on general populations, leaving a gap in understanding how these factors impact medical students specifically. By comparing our findings with those from other populations, this research aims to identify predictors of IBS that are pertinent to the UAE context. Additionally, this study may encourage medical schools to recognize the implications of psychological stress on student health and explore strategies to alleviate its negative effects.

## Materials and methods

In 2019, a cross-sectional study was conducted at the University of Sharjah (UoS), UAE, among undergraduate students enrolled in medicine, dentistry, pharmacy, and health sciences colleges. The study aimed to recruit participants aged above 18 years, from foundation to graduation year, who were randomly selected to complete a structured self-assessment questionnaire anonymously after providing consent. The sampling strategy employed a multistage stratified random sample approach, which involved stratifying the target population based on gender, college, and year of study. The exclusion criteria were defined to eliminate individuals with pre-existing gastrointestinal disorders, such as autoimmune inflammatory bowel disease, which could confound the study's results and provide an inaccurate representation of IBS prevalence among medical students. The study identified eight red-flag items that were considered exclusion criteria. 

The study identified eight red-flag items that were considered exclusion criteria [16,17]. Participants reporting one or more of these red-flag items were excluded from the study. Exclusion criteria included nonhealing or complex perianal fistula or abscess or perianal lesions (apart from hemorrhoids), first-degree relative with confirmed IBD, weight loss (5% of usual body weight) in the last three months, chronic abdominal pain (>3 months), nocturnal diarrhea, mild fever in the last three months, presence of postprandial abdominal pain (occurring 30-45 minutes after meals), particularly following the consumption of vegetables, and presence of rectal urgency.

The sample size of 474 was calculated using a formula for determining sample size based on the anticipated prevalence of IBS in the population. Students were invited to participate in the time span of a full semester, starting 19/1/2019. The sample size was calculated considering a design effect to account for the multistage stratified random sampling approach, ensuring that the resulting sample size adequately reflects the clustered nature of the sampling method. It was calculated according to the following established formula for the determination of sample size [5].

$n = \frac{{z^2 \cdot p \cdot q}}{{d^2}}$

n=the minimum sample size, z=constant (1.96). We assumed a prevalence of 14% based on a previous study [6]. p=0.14 and q=1−p=0.86.

The Research Ethics Committee at the University of Sharjah, UAE, has given approval for the study and the data collection sheet. The questionnaire used in the study was designed with 27 questions that were divided into five segments: demographics, perceived stress scale, hospital anxiety, and depression scale, food frequency questionnaire, and ROME IV criteria (Tables 8-13). To ensure the validity and reliability of the questionnaire, established measures such as the 4-item perceived stress scale (PSS-4), hospital anxiety and depression scale (HADS), and Rome IV were incorporated from previous studies [18-20]. Additionally, four open-ended questions were included alongside close-ended questions for further exploration. For the categorization of prevalence, the following cut-off points were used: low prevalence: 1% to 10%, moderate prevalence: 11% to 20%, and high prevalence: above 20%. The questionnaire was pre-tested with a small group of students to assess clarity and relevance before being administered to the study population.

The HADS is a standardized, valid, and reliable self-report rating scale. It consists of 14 items: seven for anxiety (HADS-Anxiety) and seven for depression (HADS-Depression). It was answered using a 4-point Likert scale ranging from 0 (not present) to 3 (considerable). Additionally, the Food Frequency Questionnaire (FFQ) is a questionnaire used to obtain frequency and, in some cases, portion size information about food and beverage consumption over a specified period, typically the past month or year. The food frequency questionnaire was designed to minimize recall bias by including specific time frames for consumption (never or less than once/month, once a week, 2-4 per week, 5-6 per week). IBS was defined according to Rome IV criteria as recurrent abdominal pain on average at least one day/week in the last three months, associated with two or more of the following criteria: related to defecation, associated with a change in frequency of stool, associated with a change in form (appearance) of stool. Criteria needed to be fulfilled for the last three months with symptom onset at least six months prior to diagnosis. All instruments used demonstrated high internal consistency and reliability.

The data in the study was coded, entered, and analyzed using IBM Corp. Released 2015. IBM SPSS Statistics for Windows, Version 23.0. Armonk, NY: IBM Corp. Univariate analyses were conducted, which included descriptive statistics such as frequency and relative frequency, measures of central tendency (mean, median, and mode), and measures of variability (standard deviation), as appropriate to the type of data analyzed. Additionally, bivariate analysis was performed to study the relationship between variables. Inferential statistics tests, including Chi-square, t-test, and Pearson correlation as appropriate to the type of variables involved. The level of significance was set at 5%. 

## Results

Four hundred and seventy-one medical field students took part in the study. 324 (69.1%) were female. The mean age of respondents was 20.1 ± 1.5 years. Two hundred and fifteen (45.6%) lived in dormitories. Based on the Rome IV criteria, 82 students fell under ‘Suspected case of IBS’, with a corresponding prevalence of 17.3%. Further ahead, the parameters across which IBS prevalence was studied will be mentioned along with their significance.

Gender and IBS prevalence

Out of the participants, 69.1% (N=324) were female, while 30.9% (N=145) were male. For gender, the results indicated that 13.8% (N=20) of males had a ‘suspected case of IBS’ in comparison to 19.1% (N=62) of females (Table 1).

**Table 1:** Gender disparities in suspected cases of irritable IBS p-value = 0.159. Considered insignificant as p > 0.05. Count represented as N. Percentage and percentage of both genders as %.

			Suspected Case of IBS	Not Suspected to Have IBS	Total
What Is Your Gender?	Male	Count	20	125	145
		Percentage	13.8%	86.2%	100.0%
		Percentage of both genders	4.3%	26.7%	30.9%
	Female	Count	62	262	324
		Percentage	19.1%	80.9%	100.0%
		Percentage of both genders	13.2%	55.9%	69.1%

Academic major and IBS prevalence

Within majors’ medicine recorded the highest prevalence with 20.1% (N=45) of medical students falling under ‘suspected case of IBS’, 16.3% (N=17) of health science students, 14.3% (N=6) of pharmacy students, and finally the least prevalence was among dentistry students with 13.6% (N=14) which is illustrated in (Table 2).

**Table 2:** Prevalence of IBS across academic majors p-value = 0.469. Considered insignificant as p > 0.05. Count represented as N. Percentage and percentage of all majors as %.

			Suspected Case of IBS	Not Suspected to Have IBS	Total
What Is Your Academic Major?	Medicine	Count	45	179	224
		Percentage	20.1%	79.7%	100%
		Percentage of all majors	9.5%	37.8%	47.4%
	Pharmacy	Count	6	36	42
		Percentage	14.3%	85.7%	100.0%
		Percentage of all majors	1.3%	7.6%	8.9%
	Dentistry	Count	14	89	103
		Percentage	13.6%	86.4%	100.0%
		Percentage of all majors	3.0%	18.8%	21.8%
	Health sciences	Count	17	87	104
		Percentage	16.3%	83.7%	100.0%
		Percentage of all majors	3.6%	18.4%	22.0%

Academic year and IBS prevalence

Pertaining to the academic year, the study revealed that IBS was most prevalent among year four students with 23.3% (N=17), followed by year two students with 17.9% (N=25) (Table 3). Notably, year four is considered the final year for health science students, making it stressful for many.

**Table 3:** Prevalence of IBS across academic years p-value = 0.696. Considered insignificant as p > 0.05. Count represented as N. Percentage and percentage of both genders as %.

			Suspected Case of IBS	Not Suspected to Have IBS	Total
What Is Your Year of Study?	Foundation	Count	7	36	43
		Percentage	16.3%	83.7%	100%
		Total percentage	1.5%	7.6%	9.1%
	Year 1	Count	13	72	85
		Percentage	15.3%	84.7%	100.0%
		Total percentage	2.7%	15.2%	17.9%
	Year 2	Count	25	115	140
		Percentage	17.9%	82.1%	100.0%
		Total percentage	5.3%	24.3%	29.5%
	Year 3	Count	18	95	113
		Percentage	15.9%	84.1%	100.0%
		Total percentage	3.8%	20.0%	23.8%
	Year 4	Count	17	56	73
		Percentage	23.3%	76.7%	100.0%
		Total percentage	3.6%	11.8%	15.4%
	Year 5	Count	2	18	20
		Percentage	10.0%	90.0%	100.0%
		Total percentage	0.4%	3.8%	4.2%

Exercise and IBS prevalence 

This study didn’t reveal a significant difference in IBS prevalence (p=0.581) among those who exercise 16.3% (n=37) and those who don’t 18.2% (n=45). However, there seems to be a slight protective measure for those who exercise (Table 4).

**Table 4:** Percentages of suspected IBS between exercising participants and non-participants p-value = 0.581. Considered insignificant as p > 0.05. Count represented as N. Percentage and percentage of total as %.

			Suspected Case of IBS	Not Suspected to Have IBS	Total
Do Your Exercise	Yes	Count	37	190	227
		Percentage	16.3%	83.7%	100%
		Percentage of total	7.8%	40.1%	47.9%
	No	Count	45	202	247
		Percentage	18.2%	81.8%	100.0%
		Percentage of total	9.5%	42.6%	52.1%

Smoking and IBS prevalence

With respect to smoking, the study revealed an IBS prevalence of 4% (n=1) among the ‘Light smoker’ group when compared to 28.6% (n=4) prevalence among the ‘Average smoker’ group. The percentage difference is alarming and carries a statistical significance of (p-value: 0.028) (Table 5).

**Table 5:** Prevalence of IBS in light and average smoker groups p-value = 0.047. p-value is considered significant at p < 0.05. Count is represented as N. Percentage of light, average, and total smokers as %.

			Suspected Case of IBS	Not Suspected to Have IBS	Total
How Many Cigarettes Per Day?	Light smoker (less than 10 cigarettes/day)	Count	1	24	25
		Percentage of light smokers	4.0%	96.0%	100%
		Percentage of total smokers	2.6%	61.5%	64.1%
	Average smoker (11-19 cigarettes/day)	Count	4	10	14
		Percentage of average smokers	28.6%	71.4%	100.0%
		Percentage of total smokers	10.3%	25.6%	35.9%

Chicken consumption and IBS prevalence

The study compared different food categories and the prevalence of IBS across each of them, out of the different food consumption categories, Chicken consumption held a slight significance, with which an increased consumption of about 2-4 times and 5-6 times per week showed a lower prevalence of IBS when compared to ‘no consumption’ or a ‘once a week’ consumption, indicating a protective effect with increased consumption of chicken (Table 6). The results revealed a statistical significance of (p-value: 0.002). Based on the analysis, the proportion of suspected IBS cases appears to decrease with increased chicken consumption. This trend suggests a potential protective effect. Additionally, a chi-square test indicates a statistically significant association between chicken consumption and IBS status, reinforcing this observation.

**Table 6:** Impact of chicken consumption on IBS prevalence p-value = 0.002. p-value is considered significant at p < 0.05. Count represented as N. Percentage and percentage of all frequencies as %.

			Suspected Case of IBS	Not Suspected to Have IBS	Total
Chicken consumption	Never / less than once a month	Count	5	17	22
		Percentage	22.7%	77.3%	100.0%
		Percentage of all frequencies	1.1%	3.6%	4.7%
	Once a week	Count	21	42	63
		Percentage	33.3%	66.7%	100.0%
		Percentage of all frequencies	4.5%	8.9%	13.4%
	2-4 a week	Count	37	240	277
		Percentage	13.4%	86.6%	100.0%
		Percentage of all frequencies	7.9%	51.1%	58.9%
	5-6 a week	Count	19	89	108
		Percentage	17.6%	82.4%	100.0%
		Percentage of all frequencies	4.0%	18.9%	23.0%

PSS score and IBS prevalence

Perceived Stress Scale (PSS), a classic stress assessment instrument, was administered to the students to assess their stress levels and further subgroup them into ‘low’ and ‘high’ PSS scores. The results revealed a lower IBS prevalence of 11.9% (N=32) within the ‘low PSS score’ and 24.6% (N=50) within the ‘high PSS score.’ This significant difference was also demonstrated statically with a (P<0.001) in Table 7.

**Table 7:** IBS prevalence among students based on perceived stress levels (PSS) scores p-value < 0.0005. p-value is considered significant at p < 0.05. PSS score ranges from 4 to 20. It was considered low for 4 to 11 points and high for 12 to 20 points. Count represented as N. Percentage and percentage of all scores as %.

			Suspected Case of IBS	Not Suspected to Have IBS	Total
PSS Grouped	Low PSS score	Count	32	238	270
		Percentage	11.9%	88.1%	100.0%
		Percentage of all scores	6.8%	50.3%	57.1%
	High PSS score	Count	50	153	203
		Percentage	24.6%	75.4%	100.0%
		Percentage of all scores	10.6%	32.3%	42.9%

HADS anxiety score and IBS prevalence

The participants' anxiety scores, as measured by the HADS scale, ranged from 0 to 20, with a mean score of 9.46. Among the participants, 32.1% fell within the normal anxiety range, 29% were classified as borderline abnormal, and 38.9% were categorized as abnormal (Table 8).

**Table 8:** IBS prevalence among students based on HADS anxiety score p-value < 0.0005. p-value is considered significant at p < 0.05. HADS anxiety score ranges from 0 to 21. Scoring system: 0-7: Normal, 8-10: Borderline Abnormal, 11-21: Abnormal. Count represented as N. Percentage and percentage of all scores as %.

-			Suspected Cases of IBS	Not Suspected to Have IBS	Total
HADS Anxiety Score	Normal	Count	10	142	152
		Percentage	6.80%	93.20%	100%
		Percentage of all frequencies	2.10%	30%	32.10%
	Borderline	Count	15	122	137
		Percentage	10.60%	89.40%	100%
		Percentage of all frequencies	3.20%	25.80%	29%
	Abnormal	Count	58	126	184
		Percentage	31.60%	68.40%	100%
		Percentage of all frequencies	12.30%	26.60%	38.90%

The prevalence of suspected IBS increased with higher anxiety levels, from 6.8% in the normal group to 10.6% in the borderline abnormal group, and 31.6% in the abnormal group. The Chi-square test indicated a statistically significant association between anxiety levels and IBS prevalence (p < 0.0005).

These findings suggest a positive association between increased anxiety levels and the prevalence of IBS, highlighting that participants with higher anxiety scores were more likely to be suspected of having IBS.

HADS depression score and IBS prevalence

The participants' depression scores, as measured by the HADS scale, ranged from 0 to 19, with a mean score of 6.01. Among the participants, 65.9% fell within the normal depression range, 22.7% were classified as borderline abnormal, and 11.4% were categorized as abnormal (Table 9).

**Table 9:** IBS prevalence among students based on HADS Depression score p-value = 0.055. Considered insignificant as p > 0.05. HADS depression score ranges from 0 to 21. Scoring system: 0-7: Normal, 8-10: Borderline Abnormal, 11-21: Abnormal. Count represented as N. Percentage and Percentage of all scores as %.

-			Suspected Cases of IBS	Not Suspected to Have IBS	Total
HADS Depression Score	Normal	Count	46	266	312
		Percentage	14.60%	85.40%	100%
		Percentage of all frequencies	9.70%	56.20%	56.90%
	Borderline	Count	26	81	107
		Percentage	24.00%	76.00%	100%
		Percentage of all frequencies	5.50%	17.10%	23%
	Abnormal	Count	12	42	54
		Percentage	23.10%	76.90%	100%
		Percentage of all frequencies	2.50%	8.90%	11.40%

The prevalence of suspected IBS increased with higher depression levels, from 14.6% in the normal group to 24% in the borderline abnormal group and 23.1% in the abnormal group. The Chi-square test did not indicate a statistically significant association between depression levels and IBS prevalence (p = 0.055).

These findings suggest a trend of increased prevalence of IBS with higher depression levels, although the association was not statistically significant.

## Discussion

In this research, we aimed to investigate the prevalence of IBS, which illustrated great variation among different investigations. A systematic review assessing the global prevalence of IBS among adults revealed a wide range of mean prevalence among individual countries, from 1.1% in France and Iran to 35.5% in Mexico. Regional prevalence rates also varied significantly, with Latin America reporting the highest prevalence of 17.5% and the Middle East and Africa reporting the lowest at 5.8% [21]. Another systematic review and meta-analysis focused on global variations in IBS prevalence, specifically considering studies that utilized the more recent iterations of the Rome criteria (Rome IV) and a relatively uniform methodology. Despite these criteria, significant variance in prevalence rates still existed between countries [22]. In this light, it would be of wisdom to restrict ourselves to studies conducted within our own region.

Focusing on studies conducted within our region, a cross-sectional study among the general population of Saudi Arabia showed an IBS prevalence of 18.2% [23]. Additionally, a study targeting medical students and interns at King Abdulaziz University in Saudi Arabia found a higher prevalence of 31.8% [15]. In our study, the prevalence rate among medical students at the University of Sharjah was 17.3%, which is notably higher than the 5.8% prevalence rate reported for the general population in the Middle East and Africa. This discrepancy may be explained by the unique stressors experienced by medical students, which are distinct from those faced by the general population.

In our study, along with several other studies, we found a significant association between anxiety and a higher prevalence of irritable bowel syndrome (IBS). A comprehensive meta-analysis indicated a global prevalence rate of anxiety among medical students to be 33.8% [24], thereby emphasizing the need to investigate this factor further. The substantial evidence gathered through these studies sheds light on the impact of anxiety on IBS. A previous study conducted in Pakistan utilizing the Rome III criteria and the "Generalized Anxiety Disorder Questionnaire" yielded results that align with the expected association between anxiety and IBS [25]. Our study also corroborates these findings, revealing that 31.6% of individuals with anxiety in our population met the Rome IV criteria for IBS. Furthermore, the increased occurrence of IBS symptoms in the borderline anxiety group compared to the normal group further supports this correlation. Although participants in the depressed group demonstrated IBS symptoms 23.1% of the time, this was statistically insignificant. We hypothesize, using our data, that anxiety has a much stronger role to play in IBS when compared with depression.

Although various aspects of anxiety appear to be related to IBS, specific anxiety concerning visceral sensations emerges as the most significant factor. Additionally, we explored the role of worry and stress in the bio-psychological correlation and found evidence supporting the alternative hypothesis, suggesting that higher levels of perceived stress are associated with a greater likelihood of having IBS. Our study indicated that 24.6% of individuals in the high Perceived Stress Scale (PSS) score group were suspected of having IBS. This finding reinforces previous research that examined the influence of chronic worry and stress on irritable bowel syndrome, highlighting prominent associations between IBS and a higher frequency of generalized anxiety disorder (GAD) as well as greater levels of worry, neuroticism, anxiety sensitivity, and visceral anxiety [26]. These factors are considered strong predictors, especially among medical students who face consistently high levels of stress.

Gender was hypothesized to be directly associated with the occurrence of irritable bowel syndrome (IBS). Numerous studies have consistently reported higher rates of IBS in females, highlighting it as a significant predictor. A systematic review and meta-analysis revealed an odds ratio (OR) of 1.46 for IBS in women compared to men [22]. The potential gender contrast in IBS prevalence may be attributed to the differences in how both genders manage stress [27]. Additionally, the influence of the Microgenderome, which involves the interplay between sex hormones and their impact on gut microbiota and regulatory mechanisms in the brain, could also contribute to the observed gender differences [28]. Although we observed a higher prevalence of IBS in females, this result was statistically insignificant unless validated with a larger population sample.

Numerous studies have been done to understand the etiology of IBS, and diet emerges as a prominent factor [29]. Common dietary triggers for IBS include alcohol, caffeine, spicy foods, and fat [30]. Coffee, infamous for irritating the gastrointestinal tract, is attributed to its acidic nature [31]. It stimulates rectosigmoid motor activity, which manifests as a laxative effect [32]. Alcohol has been shown to affect GI tract motility, absorption, and permeability, especially with binge drinking (more than four drinks per day) [33].

The consumption of spicy foods, particularly those containing chili, has consistently shown an association with increased IBS symptoms [34]. As of late, huge cross-sectional research among Iranian adults illustrated a significant increase in the likelihood of IBS in females who consume spicy food at least ten times weekly, although this was not observed in males [35]. Our results, however, did not point towards a potential link of IBS to spicy food, as statistical significance was not achieved in our population. This could be attributed to the limited prevalence and preference for spicy foods where the research was conducted, limiting the availability of cases to assess. 

Concerning dietary fat, duodenal lipids influence symptoms by repressing small bowel motility and impending intestinal gas release, leading to gas retention and bloating [36]. Furthermore, duodenal lipids have also been linked to increased colorectal extreme touchiness with a heightened perception of rectal distention in IBS patients [37]. All types of food mentioned were assessed, and the most significant link established in our results was the highest susceptibility of having IBS in the group who consumed chicken once a week. Strikingly, as the frequency of having chicken increased, lower percentages of suspected IBS cases were evident, suggesting that moderate chicken consumption may exhibit protective effects.

The relationship between smoking and irritable bowel syndrome (IBS) has been a subject of interest in various studies. However, it is essential to note that the scientific understanding of this relationship remains inconclusive and complex. In our research, 28.6% of average smokers were suspected to have IBS when compared to 4% of light smokers; this finding has suggested a potential association between smoking and an increased risk of developing IBS. This result was like the results of other articles, like the study that was done in Jeddah [15] and in China [38]. This may be explained by nicotine's direct impact on the digestive system or by the fact that it serves as a sign of a distressing psychological condition [39].

When IBS knowledge was further analyzed, it was shown that individuals with higher knowledge were more likely to have the condition (28% of those with strong knowledge were thought to have IBS). This can be attributed to the fact that those who have it or have symptoms of it are typically the ones who are more knowledgeable about it, or in other cases, their relatives have been diagnosed with it, which also raises the likelihood of having it as it was predicted from prior research that it has an enigmatic way of running in families [15,40]. However, 15% of those without a diagnosis had symptoms that suggested they may be affected.

The pain and distress that follow with IBS is very tormenting and can have unfortunate consequences on school grades and achievements. Stress and anxiety have the potential to prompt IBS symptoms; patients with IBS who have a concomitant psychiatric diagnosis may manifest changes in gut-related autonomic nervous system function affecting gut motility and sensation [21], whilst IBS in its own further impacts psychological health. Individuals with IBS have considerable levels of depression and anxiety when compared with controls, as presented in a systemic review including 10 studies. Medical students are exposed to great levels of stress and reduced quality of life most of the time, and one of the main aims of this study is to let the students recognize and be aware of the importance of early psychological evaluation and screening as a preventive measure of IBS since it can highly affect the students’ academic performance. It is crucial to explore and identify proper therapeutic approaches and efficient interventions for medical students to decrease the burden of this condition in such a vulnerable population. 

The unique cultural and social dynamics of the UAE significantly impact the prevalence of irritable bowel syndrome (IBS) among medical students. The fast-paced lifestyle, characterized by high academic demands and societal pressures, contributes to elevated stress and anxiety levels, which are known risk factors for IBS. Additionally, dietary habits are evolving, with traditional Emirati foods increasingly being replaced by processed and fast foods, potentially exacerbating gastrointestinal issues. Understanding these contextual factors is crucial for developing targeted interventions that address both the psychological and dietary needs of students, ultimately enhancing their overall well-being and management of IBS.

This is one of the first studies to estimate the prevalence of IBS, a very common distressing problem, and assess the associated factors and knowledge regarding it in a population in the United Arab Emirates. We aimed to touch on some factors that can trigger IBS, such as dietary habits and mental health. Understanding the roles of psychological factors like stress and anxiety, alongside dietary habits, can empower healthcare professionals to develop holistic treatment strategies that address both mental and physical health. Furthermore, our findings could contribute to public health initiatives aimed at enhancing awareness of the mental health implications related to IBS and promoting effective coping mechanisms. By fostering a deeper understanding of how these factors interact, this research could lead to the creation of support systems that improve the quality of life for individuals with IBS, while also opening avenues for future studies to validate and assess the efficacy of these integrated approaches.

Strengths

This study is one of the first to explore the prevalence and predictors of IBS among medical, dentistry, pharmacy, and health sciences students in the UAE, providing valuable insights into a relatively understudied population. The use of standardized tools such as the Perceived Stress Scale (PSS-4), Hospital Anxiety and Depression Scale (HADS), and Rome IV criteria enhances the reliability of the collected data.

Limitations

There are several limitations to consider. The study was conducted at a single institution, limiting the generalizability of the findings to other universities or the broader UAE medical student population. The majority of participants were female students from the school of medicine, which may have skewed the results and limited the representativeness of the sample. Additionally, the use of self-reported questionnaires introduces the possibility of biases, such as social desirability bias, recall errors, and subjective interpretation of questions. Furthermore, some sections of the questionnaire, such as demographics and food frequency, were custom-developed, which may impact consistency compared to fully standardized tools.

## Conclusions

The study illustrated a moderate prevalence (17.3%) of IBS was detected among medical students in UOS. Stress, anxiety, and smoking were the most significant predictors for IBS. Screening for IBS and psychological problems such as anxiety and depression among medical students is recommended. In addition, implementing stress coping strategies courses is required to enable students to trigger different stressors appropriately during their medical studies and work. This study can be the initial step that holds a significant effect towards raising awareness among the general population and educating those who are diagnosed with IBS and suspected cases that are yet not aware. In such a manner, the conduct of similar studies among the general population in the UAE is needed.

## Appendices

Appendix 1 

Consent Form for Participation in Research

**Table 10:** Consent Form for Participation in Research

Consent Form for Participation in Research
We are a group of year two medical students at the University of Sharjah conducting a research project about the prevalence and predictors of irritable bowel syndrome (IBS) among university students.
The purpose of this study is to determine the prevalence and predictors of irritable bowel syndrome among university students
You have been randomly selected to participate in this study and your participation is strictly voluntary. If you agree to participate, you will be asked to fill out a questionnaire that will take about 5-8 minutes of your time.
There are no risks associated with participation in this study. The questionnaire is anonymous, and we assure you that your responses will be confidential and will be used only for research purposes.
If you have any questions regarding this study or would like to be informed about its results, please feel free to contact Joudi Habbal at (U17101028@sharjah.ac.ae) or our research supervisor Dr. Iman Talaat (italaat@sharjah.ac.ae) For any further concerns, you may contact Dr. Suhail Al Amad, the head of the Research Ethics Committee at the University of Sharjah at 06/707304 or our research supervisor Dr. Iman Talaat (italaat@sharjah.ac.ae)
Filling out this questionnaire indicates your agreement to participate in the study.

Appendix 2: Questionnaire

Part I: Demographics

**Table 11:** Demographics

Demographics	
Question	Answer
1) What is your age?	____ years old
2) What is your gender?	I. Male
	II. Female
3) What is your nationality?	___________
4) What is your academic major?	I. Medicine
	II. Pharmacy
	III. Dentistry
	IV. Health sciences
5:) What is your year of study?	I. Foundation
	II. Year1
	III. Year 2
	IV. Year 3
	V. Year 4
	VI. Year 5
6) Are you currently living in the dormitories?	I. Yes
	II. No
7) Do you exercise?	I. Yes (answer question 8)
	II. No (skip to question 9 please)
8) How often do you exercise on average? (at least 20 min per session)	I. 1 time a week
	II. 2-3 times a week
	III. 4-5 times a week
	IV. 6-7 times a week
9) Do you smoke?	I. Yes
	II. No (skip to question 11)
10) How many cigarettes per day?	I. Light smoker (less than 10 cigarettes/day)
	II. Average smoker (between 11 and 19 cigarettes/day)
	III. Heavy smoker (more than 20 cigarettes/day)
11) What do you know about IBS (Irritable Bowel Syndrome)?	I. Never heard about it (skip question 12)
	II. Have slight knowledge
	III. Have good knowledge
12) From where did you hear about it? (you can choose more than one)	I. Diagnosed with it (firsthand experience)
	II. Family (if yes can you specify the relationship:________)
	III. Friends
	IV. Social media
	V. Awareness campaigns
	VI. Others: _________ (specify please)
13) Do you have any current diseases in your digestive system?	If yes, please specify what _____________________________
14) Do you have any previous diseases in your digestive system?	If yes, please specify what ______________________________
15) Are you on any medications?	If yes, please specify name of drug __________________________

Part II: Perceived Stress Scale 4 (PSS-4)

**Table 12:** Perceived Stress Scale 4 (PSS-4) Scoring Instructions: Total score is determined by adding together the scores of each of the four items. Questions 2 and 3 are reverse coded. Questions 1 and 4: 0 = Never; 1 = Almost never; 2 = Sometimes; 3 = Fairly often; 4 = Very often Questions 2 and 3: 4 = Never; 3 = Almost never; 2 = Sometimes; 1 = Fairly often; 0 = Very often Higher scores are correlated to more stress. [18]

Perceived Stress Scale 4 (PSS-4)	Never	Almost Never	Sometimes	Fairly Often	Very Often
1. In the last month, how often have you felt that you were unable to control the important things in your life?					
2. In the last month, how often have you felt confident about your ability to handle your personal problems?					
3. In the last month, how often have you felt that things were going your way?					
4. In the last month, how often have you felt difficulties were piling up so high that you could not overcome them?					

Part III: Food Frequency Questionnaire

**Table 13:** Food frequency questionnaire

FOOD AND AMOUNTS	AVERAGE WEEKLY CONSUMPTION			
	Never or less than once/month	Once a week	2-4 per week	5-6 per week
a. Meat, Fish, Chicken				
a1. Meat				
a2. Chicken				
a3. Fish				
b. Dairy Products				
b1. Milk, cheese, yogurt, ice cream				
c. Fast Food				
d. Spicy Food				
d1. Green & Chili Peppers				
d2. Spicy flavouring				
e .Fruits				
e1. Apples, Pears, Dried fruits				
e2. Others				
f. Vegetables				
f1. Cauliflower, Broccoli, Asparagus, Coleslaw				
f2. Others				
g. Drink				
g1. Coffee				
g2. Tea				

Part IV: Rome IV Criteria

**Table 14:** Rome IV criteria IBS was defined according to Rome IV criteria as Recurrent abdominal pain on average at least 1 day/week in the last 3 months, associated with two or more of the following criteria: 1. related to defecation 2. associated with a change in frequency of stool 3. associated with a change in form (appearance) of stool Criteria needed to be fulfilled for the last 3 months with symptom onset at least 6 months prior to diagnosis. [20]

Rome IV criteria	
Question	Answer
1. In the past 3 months have you experienced any abdominal pain?	I. Yes
	II. No
2. If yes, what was the frequency of occurrence?	I. Once per month
	II. More than once per month
3. Have you noticed any change in stool passage frequency?	I. Yes
	II. No
4. Have you noticed any change in the consistency of stool?	I. Yes (Lumpy/Hard, Loose/Watery)
	II. No
5. Have you experienced any straining and/or urgency?	I. Straining
	II. Urgency
	III. Both
	IV. None
6. Have you experienced any abdominal bloating?	I. Yes
	II. No

Part V: Hospital Anxiety and Depression Scale (HADS)

**Table 15:** Hospital Anxiety and Depression Scale Total score Depression (D) _______ Anxiety (A) _______ 0-7 = Normal 8-10 = Borderline abnormal (borderline case) 11-21 = Abnormal (case) [19]

D	A		D	A	
		I feel tense or ‘wound up’:			1 feel as if I am slowed down:
	3	Most of the time	3		Nearly all the time
	2	A lot of the time	2		Very often
	1	From time to time, occasionally	1		Sometimes
	0	Not at all	0		Not at all
		I still enjoy the things I used to enjoy:			I get a sort of frightened feeling like 'butterflies' in the stomach:
3		Definitely as much		3	Not at all
2		Not quite so much		2	Occasionally
1		Only a little		1	Quite often
0		Hardly at all		0	Very often
		I get a sort of frightened feeling as if something awful is about to happen:			I have lost interest in my appearance:
	3	Very definitely and quite badly	3		Definitely
	2	Yes, but not too badly	2		I don't take so much care as I should
	1	A little, but it doesn't worry me	1		I may not take quite as much care
	0	Not at all	0		I take just as much care as ever
		I can laugh and see the funny side of things:			I feel restless as if I have to be on the move:
3		As much as I always could		3	Very much indeed
2		Not quite so much now		2	Quite a lot
1		Definitely not so much now		1	Not very much
0		Not at all		0	Not at all
		Worrying thoughts go through my mind:			I look forward with enjoyment to things:
	3	A great deal of the time	3		As much as ever I did
	2	A lot of the time	2		Rather less than I used to
	1	From time to time but not too often	1		Definitely less than I used to
	0	Only occasionally	0		Hardly at all
		I feel cheerful:			I get sudden feelings of panic:
3		Not at all		3	Very often indeed
2		Not often		2	Quite often
1		Sometimes		1	Not very often
0		Most of the time		0	Not at all
		I can sit at ease and feel relaxed:			I can enjoy a good book or radio or TV program:
	3	Definitely	3		Often
	2	Usually	2		Sometimes
	1	Not often	1		Not often
	0	Not at all	0		Very seldom
